# Optimisation of rocker sole footwear for prevention of first plantar ulcer: comparison of group-optimised and individually-selected footwear designs

**DOI:** 10.1186/s13047-017-0208-3

**Published:** 2017-07-06

**Authors:** Stephen J. Preece, Jonathan D. Chapman, Bjoern Braunstein, Gert-Peter Brüggemann, Christopher J. Nester

**Affiliations:** 1 Centre for Health Sciences Research, University of Salford, Salford, Manchester, M6 6PU UK; 2 Institute of Biomechanics and Orthopaedics, German Sport University Cologne, Am Sportpark Muengersdorf 6, 50933 Cologne, Germany; 3 German Research Centre for Elite Sport, German Sport University Cologne, Am Sportpark Muengersdorf 6, 50933 Cologne, Germany; 4 Centre for Health and Integrative Physiology in Space, German Sports University Cologne, Am Sportpark Muengersdorf 6, 50933 Cologne, Germany

**Keywords:** Diabetes, Diabetic footwear, Therapeutic footwear, Personalised footwear, Rocker shoes, Plantar pressure, Ulceration

## Abstract

**Background:**

Appropriate footwear for individuals with diabetes but no ulceration history could reduce the risk of first ulceration. However, individuals who deem themselves at low risk are unlikely to seek out bespoke footwear which is personalised. Therefore, our primary aim was to investigate whether group-optimised footwear designs, which could be prefabricated and delivered in a retail setting, could achieve appropriate pressure reduction, or whether footwear selection must be on a patient-by-patient basis. A second aim was to compare responses to footwear design between healthy participants and people with diabetes in order to understand the transferability of previous footwear research, performed in healthy populations.

**Methods:**

Plantar pressures were recorded from 102 individuals with diabetes, considered at low risk of ulceration. This cohort included 17 individuals with peripheral neuropathy. We also collected data from 66 healthy controls. Each participant walked in 8 rocker shoe designs (4 apex positions × 2 rocker angles). ANOVA analysis was then used to understand the effect of two design features and descriptive statistics used to identify the group-optimised design. Using 200 kPa as a target, this group-optimised design was then compared to the design identified as the best for each participant (using plantar pressure data).

**Results:**

Peak plantar pressure increased significantly as apex position was moved distally and rocker angle reduced (*p* < 0.001). The group-optimised design incorporated an apex at 52% of shoe length, a 20° rocker angle and an apex angle of 95°. With this design 71–81% of peak pressures were below the 200 kPa threshold, both in the full cohort of individuals with diabetes and also in the neuropathic subgroup. Importantly, only small increases (<5%) in this proportion were observed when participants wore footwear which was individually selected. In terms of optimised footwear designs, healthy participants demonstrated the same response as participants with diabetes, despite having lower plantar pressures.

**Conclusions:**

This is the first study demonstrating that a group-optimised, generic rocker shoe might perform almost as well as footwear selected on a patient by patient basis in a low risk patient group. This work provides a starting point for clinical evaluation of generic versus personalised pressure reducing footwear.

## Background

Van Netten et al. [1] highlighted that studies investigating “*the specific role of therapeutic footwear in preventing a first foot ulcer in at-risk patients with diabetes are lacking, and are therefore urgently needed*”. Indeed, use of appropriate footwear by people with diabetes without prior ulceration is widely advocated [2–4] and motivated by a need to reduce plantar pressures that are one of the many risk factors for ulceration [5, 6]. In addition to reducing plantar pressure, however, changes in footwear habits prior to first ulceration would allow more time for footwear related behaviour change to become permanent prior to a serious foot or limb threatening event. At that stage adherence with footwear advice or prescriptions is known to affect ulcer healing and risk of re-ulceration, but effective behaviour change is often not achieved. This was demonstrated in a recent trial which observed a significant (19%) reduction in re-ulceration at 18-month follow, but only in the subgroup with good adherence and who wore customised footwear as recommended [7]. Changes in footwear choices and use prior to first ulceration might therefore mitigate the risk of a first ulcer by reducing pressure and improve longer term adherence if ulcers do occur.

Prior to investigating the potential reduction in the risk of a first ulcer due to pressure relieving footwear as Van Netten advocates [1], it is important to optimise the design of the footwear. Indeed, following a systematic review, Bus et al. [8] called for more standardised procedures to inform the design of footwear used in ulcer prevention. To optimise an intervention it is important to have an objective measure of performance. In cases of re-ulceration, reducing plantar pressures to <200 kPa has been the target for optimising footwear design [9–12]. A corresponding pressure target does not yet exist for first ulceration, but 200 kPa forms a logical initial target. Unfortunately, use of this threshold in practice relies on the use of pressure measurement at the point of footwear provision [7], and this may not always be feasible. This is especially true prior to first ulceration, when many of the footwear choices made, and implicated in subsequent ulceration, occur in a retail rather than a health care setting.

A preferable approach would be use prefabricated footwear incorporating a standardised design (i.e. same for all patients) which is known to reduce pressures <200 kPa for the majority of individuals. This will be referred to as group-optimised footwear. However, no such group-optimised design exists at present and current evidence for footwear achieving the <200 kPa threshold relates only to footwear selected/customised using individual plantar pressure data [7, 10, 12]. We refer to this as personalised footwear. Producing personalised footwear for individual patients is expensive and unlikely to be justified prior to a first ulcer unless there are significant risk factors. Therefore, in order to meet the 200 kPa target using footwear to prevent a first ulcer, it is important to understand whether group-optimised footwear that could be mass produced might suffice or whether personalised footwear is required.

In terms of the most appropriate footwear outsole designs for pressure relief, most clinical studies have investigated shoes with some form of stiff rocker outsole [7, 10]. This design has been shown to reduce peak plantar pressures at high risk sites [13]. However, a full description of the design features of the rocker outsole, or indeed the rest of the shoe, is often limited [14]. This is important because it limits our understanding of the relationship between design features (independent variable) and changes of pressure (dependent variable), limiting our ability to optimise designs for groups of patients or individual patients. Furthermore, the degree of offloading in each anatomical area is strongly influenced by the precise geometry of the rocker outsole [15, 16]. For example, our earlier work showed that altering the rocker sole apex angle by only 10–20°, can have a critical effect on the degree of offloading under the 1st metatarsophalangeal (MTP) joint [16].

A further issue is that several previous studies investigating pressure reducing footwear intended for people with diabetes have in fact involved only healthy individuals [15, 17, 18]. There are known differences between the gait and feet of those with and without diabetes [19] and the transferability of results has not been tested. Therefore, three issues need to be addressed. Firstly, we need to better understand the systematic effect of changing different rocker shoe design features on plantar pressure. With this we could propose group-optimised designs which would be used in prefabricated footwear aimed at reducing pressure beneath the 200 kPa threshold. Secondly, we need to understand the extent to which this group-optimised footwear meets the <200 kPa target compared to personalised footwear designs. Thirdly, since previous research has often assumed results from healthy participants can be transferred to those with diabetes, we need to compare footwear effects in healthy and diabetes populations.

## Methods

### Participants

Subjects with diabetes were recruited at two sites: the University of Salford (UK) and the German Sport University. At both sites participants were identified through primary care clinics and through advertisement in the community. Inclusion criteria were age ≥ 18 and medically confirmed diagnosis of type 1 or type 2 diabetes at least 6 month prior to enrolment on the study. Exclusion criteria were any current/history of foot ulceration or any foot deformity/medical foot condition that prevented the wearing of off-the-shelf therapeutic footwear. Healthy participants were recruited via community advert and required to have no medical diagnosis of diabetes or current musculoskeletal pain. All subjects provided written consent to participate in the study after appropriate ethical approval had been obtained (UK NRES 10/H1013/32). Sensation loss in the participants with diabetes was assessed using a 10 g monofilament at 5 locations (hallux, 1st MTP, 5th Metatarsal head (MTH), 5th toe and styloid process) [20].

### Footwear and plantar pressure measurement

**Fig. 1:**
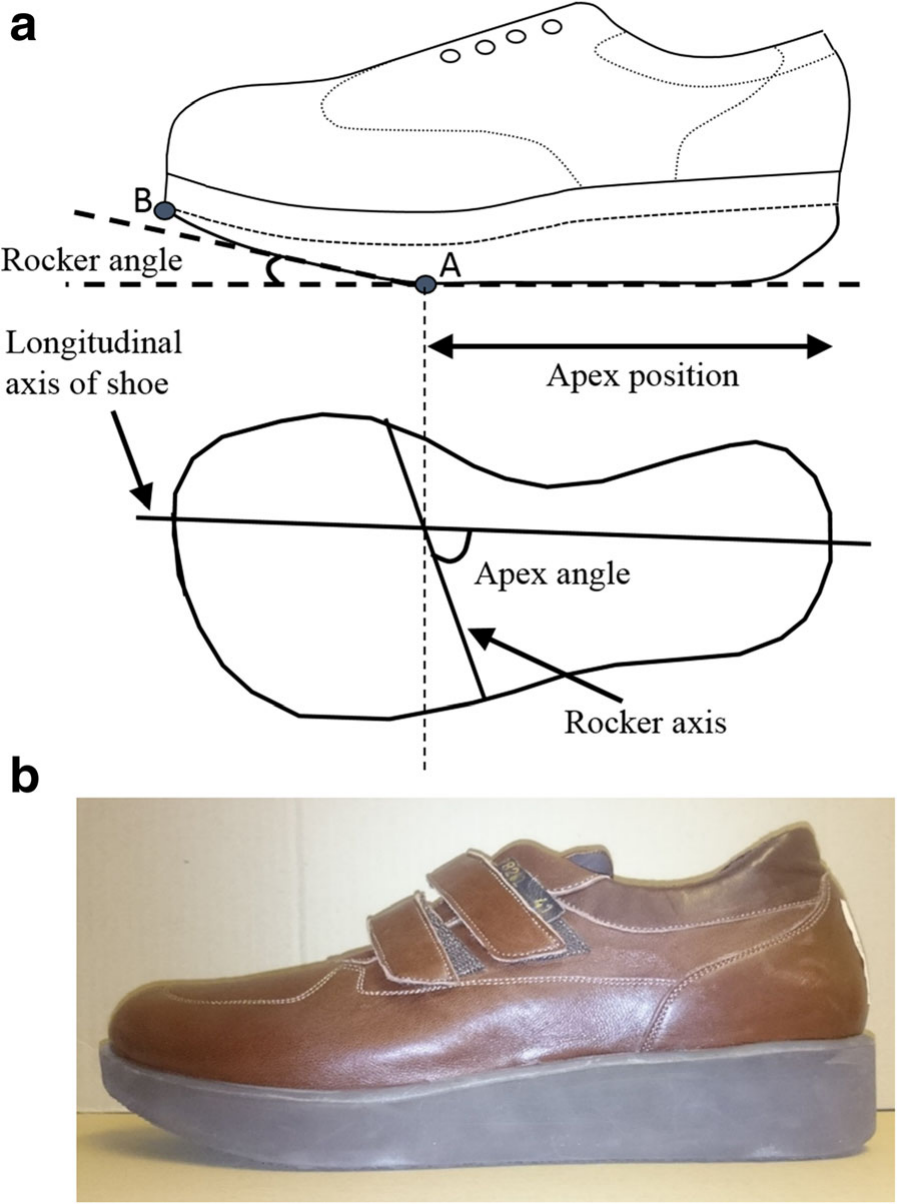
**a** Schematic to illustrate rocker angle (RA), apex position and apex angle. Apex position was varied by moving point A proximally or distally and a corresponding adjustment made to the position of point B to ensure a consistent rocker angle. **b** Example rocker shoe with RA = 20°

We sought to optimise the curved rocker outsole profile [13]. Although personalised therapeutic footwear has a wide range of different features that can be modified, this study focused on the outsole geometry of the rocker profile. This geometry can be described by three independent design features: apex angle, apex position and rocker angle [16] (Fig. 1). Apex angle and position define the orientation (relative to the long axis of the shoe) and position (% of shoe length) of a theoretical mediolateral line where the outsole begins to curve upwards under the forefoot. Our previous study demonstrated that an apex angle of 95° was appropriate for footwear designed to offload high risk regions of the forefoot [16] and was used for all footwear.

Previous research has shown that varying apex position can have a pronounced effect on peak plantar pressure [15, 16]. Furthermore, modifying apex position for each individual patient is one customisation option available when aiming to reduce plantar pressure [12]. We therefore studied apex positions of 52, 57, 62 and 67% of shoe length. The precise choice was motivated by our previous study [16] which showed marked plantar pressure increases when apex position was increased to 70% of shoe length.

Rocker angle is the angle between the floor and sole under the toe area (Fig. 1). Previous research has demonstrated that plantar pressure decreases as rocker angle is increased [15, 16] and increasing rocker angle is also a customisation option [12]. However, increasing the rocker angle from 15° to 20° has a pronounced effect on the appearance of the shoe as the thickness of the outsole must be increased. It is therefore possible that use of a 20°rocker angle would reduce adherence especially if the footwear was to be used to prevent a first ulcer (when motivation for a change in footwear habits might be lower than once ulceration has been experienced). Furthermore, our previous research suggests that the benefits of increasing rocker angle above 15° may be marginal, especially if the apex position is chosen appropriately [16]. Therefore, we studied a 15° rocker angle (aesthetic design) and a 20° rocker angle (less aesthetic design) in the hope of achieving the target pressures in the former.

A total of eight shoes were designed in which rocker angle (15° and 20°) and apex positon (52, 57, 62 and 67%) were independently varied. All footwear was manufactured with the same outsole thickness (Fig. 1) sufficient to accommodate a 20° rocker angle at all apex positions. This meant that the outsole of some designs (especially those with a 15° rocker) was unnecessarily thick but ensured that all shoes were of the same weight. In addition, a control shoe was designed with exactly the same upper as the rocker footwear but with a flexible outsole, similar to that of a running shoe [21]. The outsole of all rocker footwear was manufactured using EVA (ethyl vinyl acetate) and incorporated a 5 mm thick layer of folex which ensured that the outsole was rigid. All footwear were produced by Duna® (Italy) using CAD/CAM technology.

For each of the nine shoes, in-shoe plantar pressure was collected using Novel Pedar-X system (50 Hz) whilst participants walked at 1 m/s along a 20 m walkway. Speed was monitored during each trial using optical timing gates and only those trials within 10% of the target speed used for further analysis. Shoe order was randomised, using a custom Matlab program, and participants completed a familiarisation period of three-four minutes before data collection. A minimum of 25 steps was collected for each shoe. Following collection, the data was visually checked to identify the steps at the start and end of each walking trial which were then removed. Peak plantar pressures were calculated for each shoe design in three high-risk [10] regions: 1st metatarsophalangeal (MTP) joint, 2-4th metatarsal heads (MTH) and hallux. The Pedar sensors corresponding to each region were defined following Cavanagh et al. [22] and the peak pressures, calculated for each region, averaged across all steps to give a single value for each region and shoe. This process was repeated across all participants using custom Matlab software. The statistical analysis (outlined below) showed similar trends for both the left and right sides and therefore only data from the left side are presented in this paper.

### Statistical analysis

A two-way ANOVA model with repeated measures was used, in each anatomical region, to explore the effect of apex position and rocker angle on plantar pressure in the people with diabetes. This analysis was used to test for main effects of apex position and rocker angle and also to identify any possible interactions. If significant differences in main effects were observed, pairwise differences were investigated using a Bonferroni correction for multiple comparisons. Before testing, all data was checked for normality and homogeneity of variance. A significance level of α < 0.01 was chosen for all ANOVA analyses.

An optimal apex position was then identified, from descriptive statistics, as that which minimised pressures for the largest proportion of individuals with diabetes. This optimal position was taken to be the group-optimised design. Given our focus on two separate rocker angles (RA) and concerns over the aesthetics of the larger rocker angle, a group-optimised design was defined separately for the 15° and the 20° RA.

In order to address our second research question, we first compared peak pressures between the group-optimised design (defined above) and a personalised design. This personalised design was identified on an individual participant basis as the apex position which corresponded to the minimum peak pressures for that participant. This comparison, between group-optimised and personalised footwear, was carried out separately for each rocker angle.

We then quantified the proportion of individuals for which pressures were below the critical 200 kPa threshold in both the group-optimised design and the personalised design. Again, this was carried out separately for the two different rocker angles in each anatomical region.

Finally, in order to understand whether footwear responses were similar between people with diabetes and healthy individuals, we repeated the ANOVA analysis (described above) on the healthy control subjects. We also determined the group-optimised design for the healthy group. In addition, to compare characteristics of the two groups, the mean peak pressures (across all eight rocker shoe designs) were compared between the patients with diabetes and the healthy control group using an independent t-test.

## Results

A total of 102 individuals (52 male) with medically confirmed diagnosis of diabetes were recruited. These participant had a mean (SD) age of 57 (9 years), weight of 87 (18) Kg and height of 170 (9) cm. Loss of sensation at one foot site was identified in 25 participants with and at 2 or more sites in 17 participants (according to [20]). A further 66 healthy individuals (36 male) were recruited. There were no differences in age, 56 (8) years or height, 173 (8) cm between the healthy group and those with diabetes. However, an independent t-test showed that the healthy participants were of lower weight (*p* < 0.001, 74 (14) Kg).

**Fig. 2:**
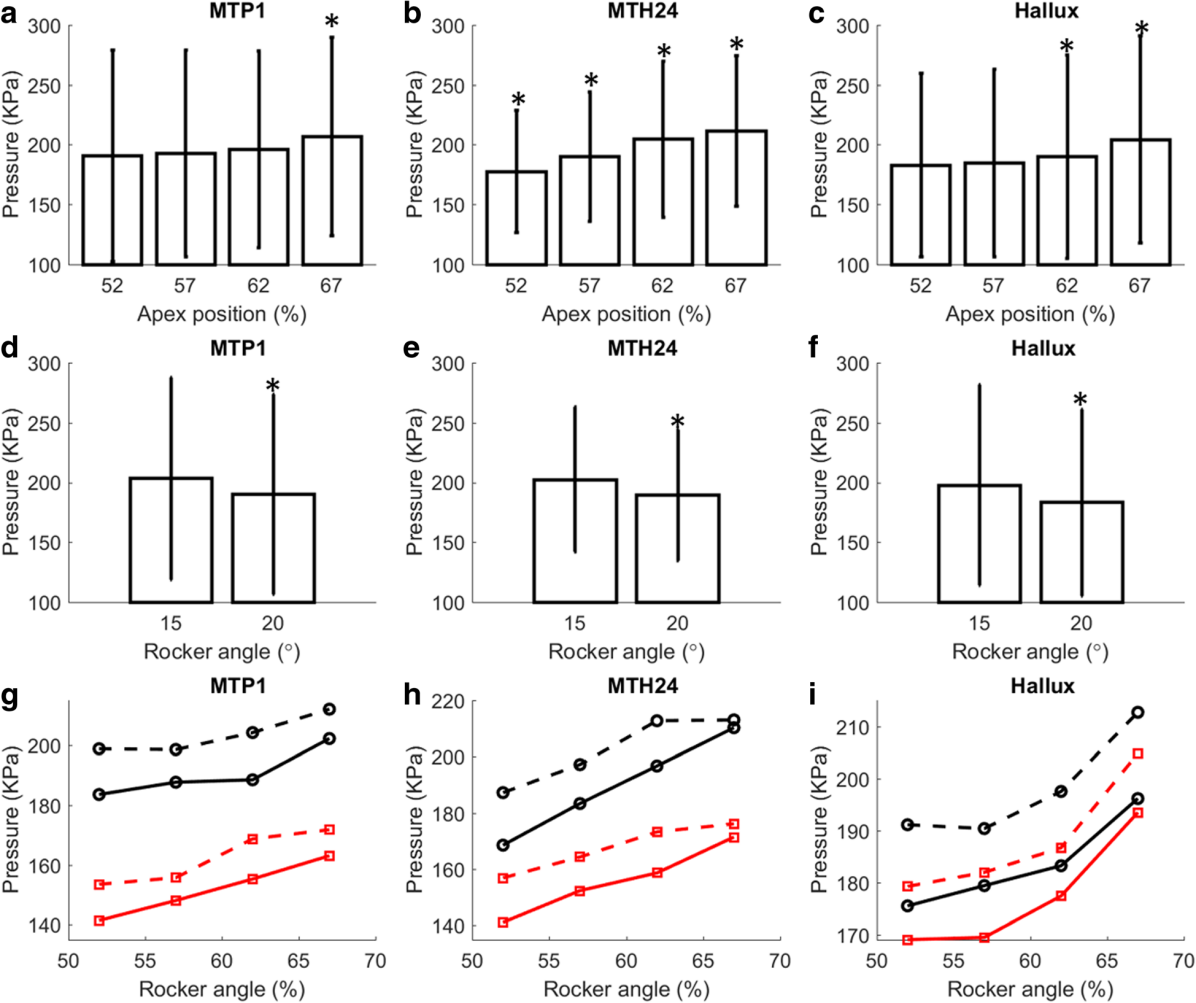
The effect of varying apex position (**a**-**c**) and rocker angle (**d**-**f**) on peak plantar pressure under the 1st MTP, 2-4th MTH and Hallux in people with diabetes (*n* = 102). The symbol * denotes a significant pairwise difference (*p* < 0.001) between a condition and at least one of the three other apex positions (plots **a**-**c**) or other rocker angle (plots **d**-**f**). The vertical lines illustrate the standard deviations. Plots g-i show the interaction between apex position and rocker angle (RA = 15° shown as dotted and RA = 20° shown as a dashed line) for the people with diabetes (black) and also for the healthy individuals (*red*)

There was a clear trend for pressure to increase as apex position was moved distally (Fig. 2a-c, Table 1). This effect was consistent across the three anatomical regions but was most pronounced in the 2-4th MTH region, for which there were pairwise differences in peak pressure between every apex position (Table 1). There was also a significant main effect of rocker angle in each anatomical region, with pressure decreasing as rocker angle was increased from 15° to 20° (Fig. 2d-f, Table 1). No interactions were observed in either the 1st MTP region or the hallux region (Table 1), showing that the effect of varying apex position was the same irrespective of rocker angle. However, in the 2-4th MTH region, increasing apex position angle from 62% to 67% lead to an increase in pressure with the 15° but not the 20° rocker angle (Fig. 2h, Table 1). Nevertheless, at the more proximal apex positions (when there was lowest pressures), the effect of changing apex position was consistent across the two rocker angles (Fig. 2h).

**Table 1:** ANOVA statistics, in each anatomical region, for the main effects of apex position, rocker angle and also for the interaction

	1st MTP	2–4 MTH	Hallux
Apex Position	F = 18.2, *p* < 0.001*	F = 157.1, *p* < 0.001*	F = 35.7, *p* < 0.001*
52°-57°	(−7.3, 3.5), *p* = 1.0	(−15.6, −9.1), *p* < 0.001*	(−6.4, 3.2), *p* = 1.0
52°-62°	(−12.2, 1.9), *p* = 0.32	(−32.9, −20.7), *p* < 0.001*	(−12.8, −1.3), *p* = 0.008*
52°-67°	(−23.4, −8.5), *p* < 0.001*	(−39.0, −28.5), *p* < 0.001*	(−28.4, −13.9), *p* < 0.001*
57°-62°	(−9.4, 2.9), *p* = 0.97	(−18.9, −9.7), *p* < 0.001*	(−11.6, 0.6), *p* = 0.10
57°-67°	(−20.7, −7.3), *p* < 0.001*	(−25.5, −17.2), *p* < 0.001*	(−26.4, −12.7), *p* < 0.001*
62°-67°	(−15.7, −5.9), *p* < 0.001*	(−10.5, −3.4), *p* < 0.001*	(−19.8, −8.3), *p* < 0.001*
Rocker Angle	F = 67.4, *p* < 0.001*	F = 179.5, *p* < 0.001*	F = 76.2, *p* < 0.001*
15°-20°	(9.8, 16.0)	(10.9, 14.7)	(11.1, 17.6)
Interaction	F = 1.5, *p* = 0.22	F = 19.1, *p* < 0.001*	F = 1.0, *p* = 0.41

Both the F-statistic and associated *p*-value have been reported. In addition, the 95% confidence intervals, and associated *p*-values, for the pairwise comparisons between different apex positions are included. Note that these *p*-values have been adjusted using a Bonferroni correction for multiple comparisons. All statistical differences (*p* < 0.01) have been marked with an *

**Fig. 3:**
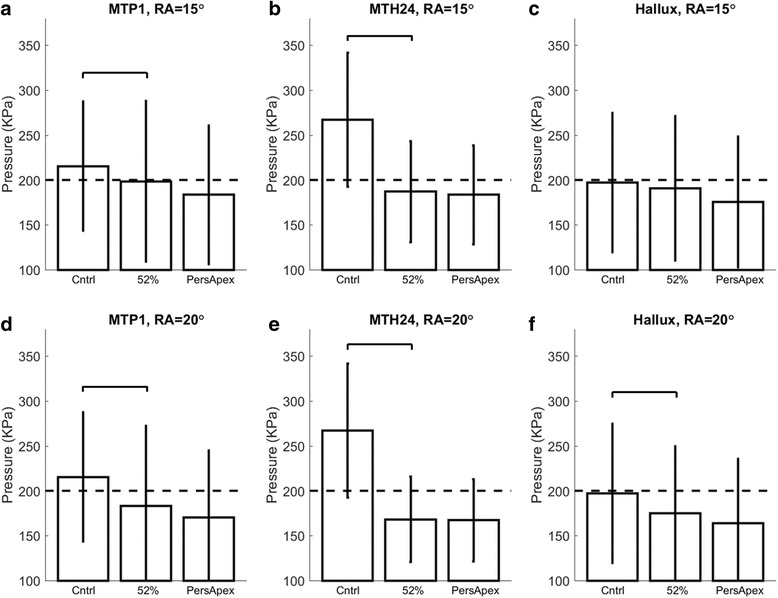
Comparison of peak plantar pressure between the control (Cntrl) shoe, the group-optimised design (apex position = 52%) and personalised apex design (PersApex) in footwear with a rocker angle (RA) = 15° (**a**-**c**) and footwear with a RA = 20° (**d**-**f**) for the three anatomical regions. The horizontal dotted line represents the 200 kPa threshold and the horizontal bars denote a significant difference between the control shoe and group-optimised design (*p* < 0.001). Diabetes participants only

The apex position at 52% of shoe length was found to minimise peak pressures for the largest proportion of individuals for both rocker angles (Table 2). The one exception was in the hallux region with a 15° rocker angle in which the 57% apex was shown to be optimal. Given the consistency of the 52% apex position, this was selected as the group-optimised design for all anatomical regions and both rocker angles. Peak pressures were significantly higher in the control shoe compared to the group-optimised designs (Fig. 3, *p* < 0.001). This difference was most pronounced for the 2-4th MTH region, for which the two group-optimised designs produced 30% and 37% reductions in peak pressure (for the RA = 15° and 20° respectively, Fig. 3).

**Table 2:** Distribution of best apex position (corresponding to minimum peak pressure) across the cohort for the two rocker angles in each of the three anatomical regions

		Participants with diabetes		Healthy individuals	
	Apex position	Rocker angle =15°	Rocker angle = 20°	Rocker angle =15°	Rocker angle = 20°
**1st MTP**	52%	**42%**	**46%**	**39%**	**56%**
	57%	30%	26%	39%	32%
	62%	20%	23%	14%	9%
	67%	8%	5%	8%	3%
**2–4 MTH**	52%	**75%**	**87%**	**68%**	**88%**
	57%	19%	13%	23%	8%
	62%	2%	0%	6%	5%
	67%	5%	0%	3%	0%
**Hallux**	52%	25%	**45%**	**33%**	**35%**
	57%	**38%**	25%	32%	41%
	62%	30%	21%	27%	15%
	67%	75	9%	8%	9%

Percentage values are provided for each apex position, with the group-optimised design (highest percentage) shown in bold. These data have been provided separately for the participants with diabetes and also the health individuals

There were only small differences in performance, between the group-optimised and personalised footwear, within the context of the 200 kPa threshold. For example, with a RA = 15°, the proportion of individuals with pressures <200 kPa was only 4–6% larger with the group-optimised design, compared to the personalised design. Similar trends were observed with the 20° rocker angle, however, interestingly, there was no increase in the proportion of participants beneath the 200 kPa threshold in the 2-4th MTH region (Table 3) with personalised footwear. Nevertheless, there was up to 12% increase in the proportion of individuals under the 200 kPa threshold when the group-optimised 15° rocker angle was compared to the group-optimised 20° rocker angle design (Table 3). Table 3 also illustrates the proportion of individuals with diabetes and peripheral neuropathy under the 200 kPa threshold. These data follow similar trends to those of the full cohort of people with diabetes, despite the fact that peak pressures (across all the eight rocker shoe designs) were between 6 and 9 kPa higher in the neuropathic (*n* = 17) compared to the non-neuropathic group (*n* = 85).

**Table 3:** The proportion of participants with a peak pressure below 200 kPa in the control shoe, the group-optimised design (52% apex) and the personalised design (individually selected apex) for both the 15° and 20° rocker angles (RA), in each of the three anatomical regions

		Below 200 kPa (RA = 15°)		Below 200 kPa (RA = 20°)	
		Participants with diabetes (*n* = 102)	Participants with diabetes and peripheral neuropathy (*n* = 17)	Participants with diabetes (*n* = 102)	Participants with diabetes and peripheral neuropathy (*n* = 17)
1st MTP	Control shoe	46%	35%	46%	35%
	Group (52% apex)	66%	65%	74%	82%
	Personalised apex	72%	71%	78%	82%
2–4 MTH	Control shoe	13%	18%	13%	17%
	Group (52% apex)	69%	53%	81%	76%
	Personalised apex	73%	53%	81%	76%
Hallux	Control shoe	60%	59%	60%	59%
	Group (52% apex)	66%	65%	71%	71%
	Personalised apex	70%	71%	75%	71%

Data are reported on all diabetes participants (*n* = 102) and also participants with diabetes and peripheral neuropathy (*n* = 17)

When the ANOVA analysis was repeated on the healthy participants, almost identical statistical trends were observed. Specially, there were main effects of apex position and rocker angle for every region with only the 2-4th MTH region showing an interaction (Fig. 2g-i). The distribution of best apex positions also followed a similar pattern to that of the group with diabetes (Table 2), illustrating the group-optimised design could also be identified from data on healthy individuals. Nevertheless, pressures were higher in the group diabetes by 25% in the 1st MTP region (*p* < 0.01) and 21% in the 2-4th MTH region (*p* < 0.01).

## Discussion

This study sought to understand the relationship between peak plantar pressure and two rocker sole design features in people with diabetes. The purpose was to use this understanding to propose a group-optimised shoe that could be used to reduce pressures below 200 kPa and potentially avoid the need for personalised footwear. The data identified that a stiff soled rocker shoe incorporating an apex position at 52% of shoe length was optimal for pressure reduction in three high-risk regions of the forefoot. Furthermore, by combing this with a 20° rocker angle, peak pressures were reduced below the 200 kPa threshold in a large proportion (71–81%) of individuals with diabetes. Importantly, the optimal apex of 52% was the same for all three high-risk plantar regions. This design configuration could therefore be appropriate for prefabricated footwear and provided without the need for plantar pressure measurement and personalisation of footwear design.

Based on requests in the literature [1] we focused on a cohort with diabetes but no history of ulceration. This relatively lower risk group are unlikely to choose footwear which they deem to have an unacceptable appearance, such as the extra-depth footwear sometimes advocated post first ulceration [23]. Therefore, we sought to understand the effect of decreasing rocker angle from 20° to 15°, as using this lower angle will produce footwear with a thinner outsole that may be perceived as more acceptable and may enhance adherence. The decrease in rocker angle from 20° to 15° led to an decrease in the number of participants beneath the critical threshold of 200 kPa (Table 3). However, decreases were modest (6–12%) for the 1st MTP and 2–4 MTH regions and small in the hallux region (5%). We therefore suggest that, if individuals are unwilling to wear a shoe with a 20° rocker angle, then a 15° could be prescribed as an acceptable alternative.

Through a series of two studies (this current study and a previously published study [16]), we have attempted to understand the combined effect of the three design features of curved rocker footwear: apex angle, apex position and rocker angle. Data from both these studies supports the idea that increasing rocker angle will decrease plantar pressures. However, there appears to be a complex relationship between apex angle and apex position. Whereas in our first study, we investigated the effect of varying apex position when apex angle was fixed at 80° [16], in this current study apex angle was fixed at 95°. Interestingly, it was not possible to specify a group-optimised apex position in the previous study because of considerable inter-subject variability. However, the use of a 95° apex angle led to a much more consistent response in this current work which supports the idea of a common footwear design for people with diabetes prior to first ulceration. This group-optimised design would incorporate an apex angle of 95°, an apex position of 52% of shoe length and a rocker angle of 15° or 20°.

We sought to understand footwear performance in the context of the 200 kPa threshold suggested by Owings et al. [9]. This threshold was suggested based on mean in-shoe pressures from individuals with a prior history of ulceration who had remained ulcer free for a prolonged period (0.4–14.4 years). The feet of those pre-first ulceration are less likely to be at comparable risk of ulceration. As such 200 kPa may be a conservative target and, if the goal is to prevent primary ulceration, this target could perhaps be increased. Re-analysis of our data with a revised threshold of 220 kPa (i.e. 10% higher threshold) demonstrated a 5–7% decrease in the proportion of feet at risk with the group-optimised design (for example the percentage under the threshold increased from 81% to 87% in the 2-4th MTH region). However, importantly, the differences in the proportion of people under the revised threshold between the group-optimised design and the personalised design were very similar to those observed with the 200 kPa threshold.

A clinical trial rather than a laboratory study is needed to test the clinical efficacy of footwear for the prevention of first ulceration. In their recent systematic review, Van Netten et al. [1] advocated evaluating interventions on the cohorts for which they are intended and, in the context of interventions to prevent first ulceration, this would involve individuals deemed at high risk of first ulceration. A limitation of this current study is that most participants would be considered low risk as they did not demonstrate sensory loss. However, our cohort did include 17 who demonstrated evidence of neuropathy and we quantified the proportion of this subgroup for whom the group-optimised rocker design reduced pressure beneath the 200 kPa threshold. These data showed very similar trends (Table 3) compared to the full cohort with diabetes. This provides some evidence that our proposed rocker design may be appropriate, or at least a good starting point, for a higher-risk population. Nevertheless, people with diabetes can also present with deformity, Charcot arthropopathy, or digit amputations, all of which will affect gait and foot function. These may therefore influence the response to footwear designs too. We acknowledge, therefore, that our proposed design may not be immediately transferrable to feet affected in different ways by diabetes.

It is important to recognise that even if the pattern of response to the footwear designs is insensitive to diabetes, elevated pressures, and neuropathy, as we suggest, the actual pressure values in people with neuropathy and at higher risk would differ to those we report. This limitation is important because the proportion of individuals over the 200 kPa would likely be higher than we report. Nevertheless, as explained, 200 kPa is likely to be a conservative target for those without prior ulceration and so the use of the group-optimised footwear design would still decrease the proportion of individuals considered at risk.

There are a number of other limitations to the current study which should be highlighted. Firstly, due to the practicalities of experimental testing, we chose to focus on a specific shoe design, varying two specific design features across a number of discrete levels. Our findings are therefore only valid for curved rocker footwear. Nevertheless, our approach of systematically varying independent design features in order to identify a group-optimised shoe design could be applied in other footwear designs. A further limitation is that we did not compare our group-optimised footwear with fully customised footwear, incorporating an extra depth upper along with a customised insole with metatarsal pads and cut outs etc. However, the aim of this study was to specify a group-optimised outsole design and the beneficial effects of other footwear modifications are likely to be additive. Such customised footwear comes at greater cost, and perhaps lower adherence, and seems out of context for the prevention of first ulceration, when most footwear is still sought via a retail route. However, our proposed group-optimised design could be used as a starting point for fully customised footwear if further reductions in pressure were necessary.

## Conclusions

By studying the relationship between footwear design features and peak plantar pressure, we have been able to suggest a group-optimised design for plantar pressure reducing footwear. Our data demonstrate that this design can reduce pressures below the 200 kPa threshold in the majority of people with elevated plantar pressure but otherwise at low risk of first ulceration. The results also show that personalised selection of footwear based on collection of plantar pressure data may offer only marginal gains in this population.

## Abbreviations

MTH: metatarsal head
MTP: metatarsophalangeal
RA: rocker angle
